# Integrating artificial intelligence in strabismus management: current research landscape and future directions

**DOI:** 10.3389/ebm.2024.10320

**Published:** 2024-11-25

**Authors:** Dawen Wu, Xi Huang, Liang Chen, Peixian Hou, Longqian Liu, Guoyuan Yang

**Affiliations:** ^1^ Department of Ophthalmology, West China Hospital, Sichuan University, Chengdu, China; ^2^ Laboratory of Optometry and Vision Sciences, West China Hospital, Sichuan University, Chengdu, China; ^3^ Laboratory of Macular Disease, West China Hospital, Sichuan University, Chengdu, China

**Keywords:** strabismus, artificial intelligence, deep learning, large model, multimodal, eye care

## Abstract

Advancements in artificial intelligence (AI) are transforming strabismus management through improved screening, diagnosis, and surgical planning. Deep learning has notably enhanced diagnostic accuracy and optimized surgical outcomes. Despite these advancements, challenges such as the underrepresentation of diverse strabismus types and reliance on single-source data remain prevalent. Emphasizing the need for inclusive AI systems, future research should focus on expanding AI capabilities with large model technologies, integrating multimodal data to bridge existing gaps, and developing integrated management platforms to better accommodate diverse patient demographics and clinical scenarios.

## Impact statement

Early diagnosis and treatment of strabismus are crucial for preventing irreversible visual impairment and improving patient outcomes. This review explores the transformative potential of AI in strabismus management, highlighting significant advancements in screening, diagnosis, and surgical planning that have improved diagnostic accuracy and surgical outcomes. It addresses current challenges such as the underrepresentation of diverse strabismus types and reliance on single-source data, emphasizing the need for inclusive AI systems integrating multimodal data. As the first dedicated review on AI’s role in strabismus, it provides valuable insights and guides future research. This new information highlights AI’s potential to enhance patient outcomes, improve ophthalmic care, and contribute to societal welfare, setting the stage for further advancements in AI applications in strabismus management.

## Introduction

Strabismus, a prevalent ocular disorder characterized by the misalignment of eyes [1], predominantly affects children, leading to rapid deterioration of binocular vision, monocular suppression, anomalous retinal correspondence, and ultimately, irreversible and permanent visual impairment. While primarily seen in children, strabismus can occur at any age, significantly impacting visual function, appearance, learning abilities, employment opportunities [2], and mental health [3], thus constituting a significant societal concern [4]. Delays in diagnosis and treatment often culminate in binocular vision dysfunction or irrevocable vision loss [5]. Given its subtle onset and diverse subtypes, early diagnosis significantly enhances recovery chances. Certain types of infantile strabismus, such as intermittent esotropia in infants under 6 months, may naturally improve by the age of one, advocating for close monitoring to circumvent premature surgical interventions [6]. In light of the above, implementing screening for high-risk groups and precisely diagnosing subtypes of strabismus, coupled with early and appropriate interventions (such as surgery), is of paramount importance. Screening refers to a binary classification process aimed at determining the presence or absence of strabismus, assisting in identifying cases that require referral for further evaluation. Diagnosis, on the other hand, involves multi-class classification to determine the specific subtype of strabismus, which is critical for deciding on appropriate treatment options. However, current strabismus screening and diagnosis are primarily conducted manually by strabismus and pediatric ophthalmologists using methods such as the corneal light reflex test and cover test, which heavily rely on patient cooperation and the physician’s skill and experience.

**FIGURE 1:**
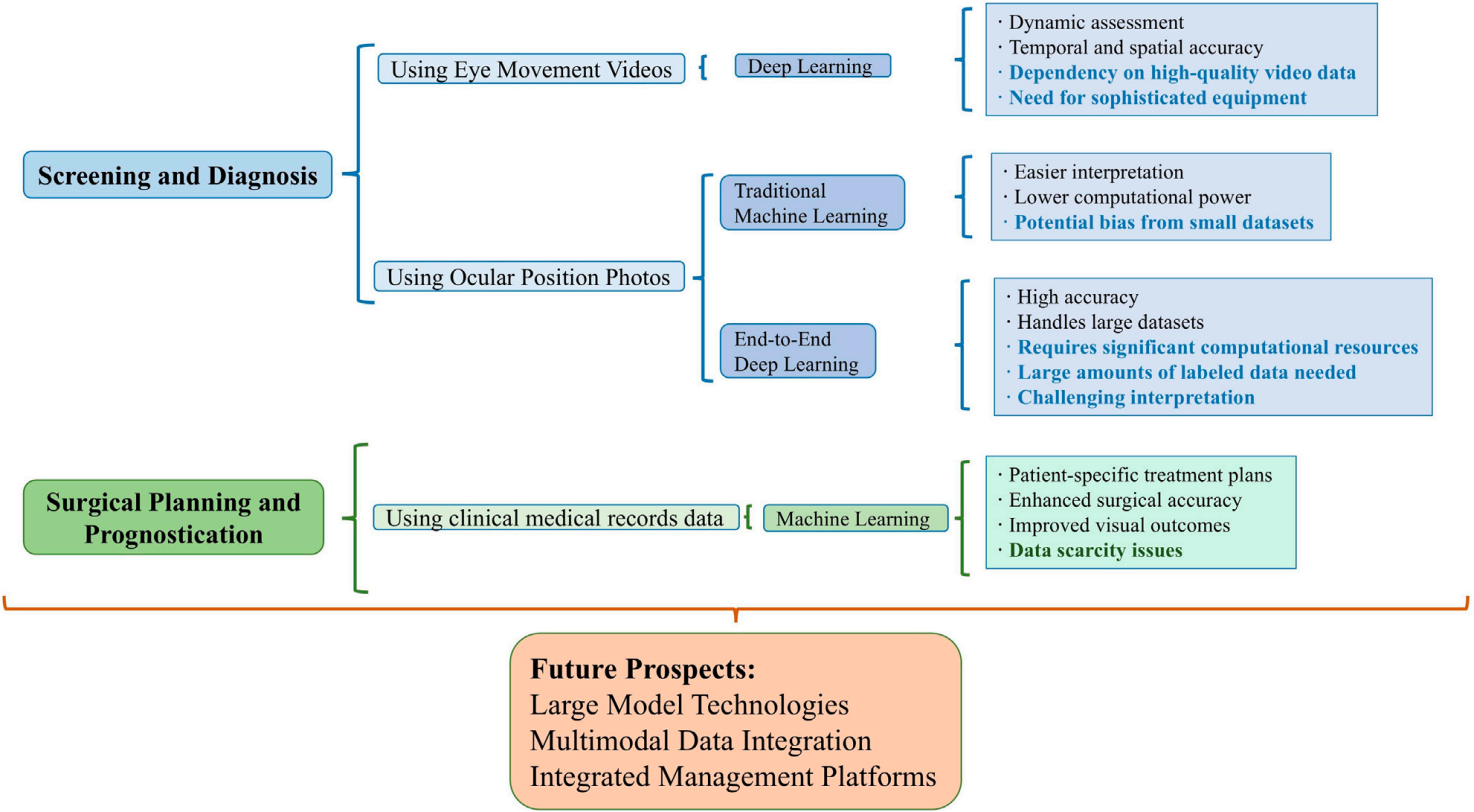
Overview of integrating AI in Strabismus management. This figure summarizes the use of different data modalities and AI algorithms in strabismus management tasks, highlighting their advantages and disadvantages. Disadvantages are indicated in bold.

Recent years have seen an exponential growth in artificial intelligence (AI) technology, encompassing research on all common ocular diseases, including anterior segment diseases like keratoconus [7] and cataracts [8], and posterior segment diseases such as retinal diseases [9] and optic nerve-related conditions [10]. The year 2023 witnessed unprecedented breakthroughs in medical AI capabilities, propelled by the transformative development of large models, such as ChatGPT [11]. Google’s Med-PaLM [12], achieving expert-level performance on U.S. medical licensing examination questions, and the publication of foundational large model articles in prestigious journals like Nature [13], have underscored the emerging landscape of medical AI large models. The substantial potential of AI has markedly improved the accuracy of ocular disease screening and diagnosis, contributing to reduced healthcare workload, lower medical costs, and addressing the shortage of ophthalmologists, thereby striving for comprehensive healthcare resource coverage and enhanced public health. Notably, AI research in strabismus treatment and prognosis prediction has also been flourishing. This article, set against the backdrop of large model intelligence in medicine, primarily outlines the current state of research on AI applications in the screening, diagnosis, surgical parameter estimation, and prognosis prediction of strabismus, and provides a perspective on its research trends. Figure 1 and Table 1 summarize the relevant studies and applications discussed.

**TABLE 1:** Summary of AI applications in strabismus management.

Authors, year	Task	Age, years	Disease type	Data type	Sample size	AI algorithm	Output
Strabismus Screening and Diagnosis							
Long 2019 [14]	Diagnosis	0–3	Visual impairment (including strabismus)	videos	4,196 subjects	Temporal Segment Networks	AUC = 0.816–0.930
Chen 2023 [15]	Screening	0–4	Visual impairment (including strabismus)	videos	3,652 subjects	EfficientNet-B4	AUC = 0.940 for internal validation AUC = 0.843 for external validation
Miao 2020 [16]	Ocular deviation measurement	>6	Exotropia	VR-based pupil tracking	17 subjects	stepwise approximation	Mean deviation of 0.4° ± 0.2° in orthotropia, 8.1° ± 5.5° in exotropia
Saisara 2017 [17]	Screening	7–50	Strabismus	Games and Eye tracking data	50 subjects	Gazepoint Analysis	Identified Dthreshold ≥0.05 for strabismus
Chen 2015 [18]	Diagnosis	3–63	Esotropia, Exotropia and Hypertropia	Eye tracking data	225 subjects	Eye-tracking with Tobii X2-60	Identified subjects’ fixation accuracy
Valente 2017 [19]	Diagnosis	—	Exotropia	Eye tracking videos	15 videos of 7 strabismic subjects	Image processing	Sensitivity = 0.800 Specificity = 1.000 Accuracy = 0.933
Chen 2018 [20]	Screening	25–63	Recessive, intermittent, and manifest strabismus	Eye tracking images	42 subjects	AlexNet VGG-F VGG-M VGG-S VGG-16 VGG-17	VGG-S: Accuracy = 0.952 Specificity = 0.960 Sensitivity = 0.941
Ma 2020 [21]	Screening	8–10	Strabismus, myopia and anisometropia	Images	100 subjects	Image processing	Accuracy = 0.940 Specificity = 0.980 Sensitivity = 0.800
Kang 2022 [22]	Diagnosis	—	Strabismus	Images	828 subjects	U-Net	Sclera Segmentation: Accuracy = 0.998 Specificity = 0.975 Sensitivity = 0.999 DSC = 0.969 Limbus Segmentation: Accuracy = 0.999 Specificity = 0.956 Sensitivity = 0.999 DSC = 0.957
Almeida 2015 [23]	Diagnosis	—	Exotropia, Esotropia, Hypertropia and Hypotropia	Images	200 images of 40 strabismic subjects	SVM	Accuracy: 0.880 (ET) 1.000 (XT) 0.803 (HT) 0.833 (HoT)
de Oliveira Simoes 2019 [24]	Diagnosis	—	Exotropia, Esotropia, Hypertropia and Hypotropia	Images	225 images of 45 strabismic subjects	U-Net, ResNet	Accuracy = 0.966 Specificity = 1.000 Sensitivity = 0.958
Mesquita 2021 [25]	Diagnosis	5–15	Exotropia, Esotropia, Hypertropia and Hypotropia	Images	224 subjects	Image processing	Accuracy = 0.845 Specificity = 0.844 Sensitivity = 0.895 Kappa coefficient = 0.430
De Figueiredo 2021 [26]	Diagnosis	6–87	Exotropia and Esotropia	Images	990 images of 110 strabismic subjects	ResNet50	Accuracy = 0.420–0.920 Precision = 0.250–0.840 Recall = 0.380–0.920 F1 = 0.290–0.880 Val_Loss = 0.085–2.210
Huang 2021 [27]	Screening	—	Strabismus	Images	60 subjects	ResNet-12	Accuracy = 0.805 Specificity = 0.768 Sensitivity = 0.842
Zheng 2021 [28]	Screening	—	Exotropia and Esotropia	Images	7,026 images for training and 277 for External Validation	VGG16, Inception-V3, Xception	Inception-V3: AUC = 0.997 Accuracy = 0.968 Specificity = 0.993 Sensitivity = 0.940
Mao 2021 [29]	Screening	1–65	Exotropia	Images	5,797 subjects	InceptionResNetV2	AUC = 0.998 Accuracy = 0.990 Specificity = 0.983 Sensitivity = 0.991
Wu 2024 [30]	Screening	1–74	Exotropia, Esotropia and Vertical deviation	Images	6,194 images	VIT_16_224	AUC = 0.994, Accuracy = 0.967, Precision = 0.980, Specificity = 0.970, Sensitivity = 0.960, F1 = 0.975
Kim 2021 [31]	Diagnosis	—	Exotropia and Esotropia	Images	2023 subjects	CNN	Accuracy = 0.667
Strabismus Surgical Planning and Prognostication							
De Almeida 2015 [32]	Surgical planning	—	Exotropia and Esotropia	Clinical data	88 patients	SVR	Average error: medial rectus muscles: 0.500 mm for recoil, 0.700 for resection lateral rectus muscles: 0.600 for recoil, 0.800 for resection
Fernando 2021 [33]	Surgical planning	0.5–65	Exotropia and Esotropia	Clinical data	153 patients	DTR, RFR, ETR	MAE:0.448 – 1.038 mm RMSE = 1.496–2.447 mm
Tang 2022 [34]	Surgical planning	—	Strabismus	Clinical data	1,076 patients	WGAN-GP + lightGBM	AUC = 0.845
Mao 2021 [29]	Surgical planning	1–65	Exotropia	Images	1,070 images	InceptionResNetV2	accuracy of ±5.5° (11.5 PD) with a bias of −0.6°
Lou 2023 [35]	Surgical Planning	17.6 ± 12.7	Inferior oblique overaction	Images	106 eyes	GAR2U-Net	ICC = 0.975
Liu 2019 [36]	Surgical Prognostication	6–12.25	Intermittent exotropia	Clinical data	132 patients	SVM	Accuracy = 0.821

AI, artificial intelligence; AUC, area under curve; DSC, dice similarity coefficient; SVM, support vector machine; ET, esotropia; XT, exotropia; HT, hypertropia; HoT, hypotropia; CNN, convolutional neural network; SVR, support vector regression; DTR, decision tree regressor; RFR, random forest regressor; ETR, extra trees regressor; MAE, mean absolute error; RMSE, root mean squared error; LightGBM, light gradient-boosting machine; PD, prism diopter; ICC, intraclass correlation coefficient. This table summarizes various studies on AI applications in strabismus management, detailing the author and year, task, data type, sample size, AI algorithm, and key performance metrics.

## AI in strabismus screening and diagnosis

### AI-driven strabismus detection using eye movement videos

The integration of videos with artificial intelligence (AI) in strabismus diagnosis represents a significant advancement in pediatric ophthalmology, offering a nuanced understanding of ocular misalignments through precise and dynamic assessment. This approach capitalizes on the temporal and spatial accuracy of eye tracking to capture subtle deviations in gaze behavior, which are often elusive in traditional examination settings. By harnessing the computational power of AI, these systems analyze complex eye movement patterns efficiently, uncovering diagnostic insights that transcend human observation. Furthermore, the ability to conduct these assessments in a non-invasive, patient-friendly manner reduces the stress associated with conventional diagnostic procedures, facilitating a more comfortable experience for pediatric patients. The amalgamation of eye-tracking technology and AI not only streamlines the diagnostic process but also enhances its accuracy, paving the way for early intervention strategies that are critical in mitigating the long-term visual consequences of strabismus.

In a groundbreaking study published in Nature Biomedical Engineering, Long et al. [14] investigated deep learning for diagnosing visually impaired infants, focusing on strabismus. Using a dataset of over 4,196 infants, they employed a temporal segment network on full-length videos to identify visual impairment patterns. The study highlighted Duane Syndrome and achieved high accuracy with area under the curve (AUC) metrics of 86.4%–93.0% for congenital conditions. This research validates deep learning’s potential in diagnosing complex strabismus and underscores video-based behavioral analysis as a non-invasive pediatric ophthalmology tool [14]. Chen et al. [15] published a multicenter study in Nature Medicine, revealing a deep learning-based screening method for pediatric ophthalmic diseases, capable of diagnosing 16 common eye conditions in children, including strabismus. The study used cartoon videos to engage children, while advanced imaging recorded their head and eye movements. The neural network analyzed gaze patterns and facial features, distinguishing specific conditions. Using a dataset of 3,652 subjects and over 25 million video frames, the model achieved an AUC of 0.940 for internal validation and 0.843 for external validation [15].

While the studies mentioned offer significant insights into screening multiple pediatric eye diseases, including strabismus, their accuracy specifically for strabismus diagnosis highlights room for improvement. Given the potential of AI and eye-tracking technologies to advance strabismus diagnosis, automating this process presents a more accurate and efficient alternative to traditional methods. Therefore, previous studies have been published that focus specifically on AI-based strabismus screening and diagnosis using eye movement videos. Miao et al. [16] innovatively apply Virtual Reality (VR) to strabismus diagnosis, introducing a VR-based system that utilizes infrared cameras for measuring ocular deviation. In clinical trials, the VR system achieved a mean deviation of 0.4° ± 0.2° in orthotropic patients and 8.1° ± 5.5° in exotropic patients, closely mirroring doctor evaluations [16]. In the study by Saisara et al. [17], a novel strabismus screening approach combining eye tracking and gaming is proposed through the integration of the Gazepoint GP3 Eye Tracker with custom games. The system was tested on 50 volunteers, effectively identifying strabismus cases with a specific threshold value [17]. Chen et al. [18]’s study employs the Tobii X2-60 eye tracker to automate strabismus diagnosis. By contrasting object positions with eye fixations, their method identifies strabismus types. Precision is ensured through a 25-point calibration test, proving effective in diagnosing conditions like hypertropia [18]. Valente et al. [19] explore automated strabismus diagnosis through digital video analysis of the cover test. The study outlines an eight-step methodology culminating in a diagnostic accuracy of 93.33% for exotropia, with a specificity of 100% and a sensitivity of 80%. Furthermore, the system reported an average error of 2.57° in deviation measurement [19]. Chen et al. [20] developed a deep learning model capable of diagnosing strabismus by leveraging six different convolutional neural networks (CNNs) - AlexNet, VGG-S, VGG-M, VGG-16, VGG-F, and VGG-19. They collected eye-tracking data as participants looked at nine specific points, represented through gaze deviation (GaDe) images, from 42 subjects for model training and verification. Among the tested networks, VGG-S emerged as the most effective, demonstrating a specificity of 0.960 and sensitivity of 0.941 [20]. These findings highlight the tremendous potential of DL algorithms based on eye movement videos in the field of strabismus screening and subtype diagnosis.

### AI-driven strabismus diagnosis using ocular position photos

While eye-tracking technology, with its spatial and temporal accuracy, excels at capturing subtle deviations in gaze behavior and leverages the AI for precise and dynamic evaluation of eye movements in both “resting” and “task” states, facilitating the screening of multiple ocular diseases and identification of certain strabismus subtypes, the collection of eye movement videos often requires specialized equipment and is more time-consuming. Additionally, video processing demands substantial computational resources, unlike the convenience and lower computational requirements of ocular position photos. Merely a camera or even a smartphone can complete the image capture process. Instant uploads can return diagnostic results within milliseconds. Therefore, specifically for strabismus, AI systems based on ocular position photos continue to play a unique and indispensable role.

In the current realm of AI research for strabismus screening and diagnosis using ocular position photos, two main approaches prevail: algorithms for key eye region segmentation based on traditional stepwise learning, and classification algorithms based on end-to-end learning.

#### Traditional machine learning algorithms

Ma et al. [21] developed a smartphone app for the rapid screening of pediatric eye diseases, including strabismus. Utilizing image processing and AI, the app analyzes photos taken in dark rooms to evaluate facial landmarks, head tilt, and eye positions. It employs shape fitting techniques to estimate corneal light reflex and red reflex contours, quickly assessing risks of strabismus, myopia, and refractive errors in just 10 s. The app’s sensitivity and specificity for detecting strabismus are 0.80 and 0.98, respectively, proving effective for early diagnosis [21]. Kang et al. [22] developed a deep learning model using U-Net architecture to detect strabismus by segmenting the cornea and scleral limbus from 828 gaze photographs across nine different gaze positions. The model demonstrated high segmentation accuracy: 0.9984 for the cornea and 0.9992 for the limbus, with Dice Similarity Coefficients of 0.9688 and 0.9571, respectively [22]. Almeida et al. [23] employed support vector machines (SVMs) for a machine learning-based diagnostic approach to strabismus. Analyzing 200 images from 40 patients with conditions like esotropias, exotropias, hypertropias, and hypotropias, their methodology involved face segmentation and eye region detection to precisely identify eye deviations. It achieved accuracy rates of 88% for esotropias, 100% for exotropias, and over 80% for vertical deviations, with errors closely matching specialist assessments [23]. de Oliveira Simoes et al. [24] adopted a similar experimental approach. Employing a private dataset of 225 images from 45 patients, the research aimed at evaluating ocular alignment by analyzing the distance between the limb centroid and a point between the eye corners, achieving an impressive accuracy of 96.6%, with a sensitivity of 95.8% and specificity of 100% [24]. Mesquita et al. [25] explored the concordance between expert ophthalmologist diagnoses and those made by an mHealth application for strabismus in 224 children aged 5–15. The app analyzed smartphone photos, incorporating steps like face segmentation, eye region detection, and alignment comparison of the Limbus center with brightness location. The app demonstrated a sensitivity of 89.47% and specificity of 84.39% at 6 PD, affirming its utility as a screening tool despite some misclassifications [25]. De Figueiredo et al. [26] developed an app based on the ResNet50 neural network to diagnose strabismus by identifying patients’ different gaze positions in photographs from 110 patients. The model, trained to recognize combinations of left and right eyes in gaze positions ranging from 1 to 9 and version classifications from −4 to +4, achieved an overall accuracy between 0.42 and 0.92 and precision between 0.28 and 0.84 [26]. Huang et al. [27] developed a strabismus screening model with frontal facial images for face and eye region identification, and refining the detection with Otsu’s binarization and the HSV color model. The model uses the least squares method to locate the pupil’s center and assesses strabismus through eye positional similarity. Additionally, utilizing the ResNet-12 network for screening on images from 60 subjects, the team achieved diagnostic accuracy, sensitivity, and specificity of 0.805, 0.768, and 0.842, respectively [27].

#### End-to-end deep learning algorithms

Zheng et al. [28] leveraged the Inception-v3 architecture to train a model for identifying referable horizontal strabismus in children’s primary gaze photos, excluding conditions like vertical and paralytic strabismus. It achieved an average AUC of about 0.99, with sensitivity at 94.0%, specificity at 99.3%, and accuracy at 96.8% in the external validation set, outperforming resident ophthalmologists [28]. Lin et al. [29] utilized the InceptionResNetV2 architecture for training a model aimed at diagnosing horizontal strabismus, deliberately excluding vertical strabismus and intermittent exotropia during the data collection phase. The training involved 1,561 photographs of horizontal strabismus (both esotropia and exotropia) and 2,496 of normal eyes. Tested on 356 horizontal strabismus and 514 normal eyes, the model achieved a sensitivity of 0.991, a specificity of 0.983, and an accuracy of 0.990 [29]. Wu et al. [30] constructed the largest corneal light-reflection photo dataset in the field to date and trained a model based on the Transformer architecture (VIT_16_224). Unlike previous studies that focused solely on pediatric patients and a limited number of strabismus subtypes, this study encompassed all age groups and a comprehensive range of strabismus subtypes, resulting in the development of the best-performing model. The VIT_16_224 architecture outperformed the models from the aforementioned studies on a shared dataset. On an independent test set, the model achieved an accuracy of 0.967, precision of 0.980, specificity of 0.960, sensitivity of 0.970, and an F1 score of 0.975, significantly improving the generalizability and practicality of the diagnostic model. Donghwan Kim et al. [31] introduced a Convolutional Neural Network (CNN)-based model for classifying strabismus into three categories: esotropia, exotropia, and normal eye alignment, leveraging a “9-photo” front view. The training set consisted of “9-photo” sets from 73 esotropia patients, 75 exotropia patients, and 72 individuals with normal alignment. Testing was conducted with “9-photo” sets from 10 patients in each group. The model achieved a final test accuracy of 66.7% [31]. These studies highlight the model’s potential to streamline early strabismus diagnosis in young children.

#### Comparison of traditional machine learning and end-to-end deep learning algorithms

In comparing traditional machine learning algorithms and end-to-end deep learning algorithms for strabismus diagnosis, several key differences and similarities emerge. Traditional machine learning algorithms typically involve a series of pre-processing steps, feature extraction, and model training phases. Common techniques include Support Vector Machines (SVM), decision trees, and regression models. For eye region segmentation, researchers often use pre-trained facial detection models to extract key regions and calculate coordinates for critical areas like the pupil center and corneal light reflex points. By comparing these results to predetermined thresholds, the presence and subtype of strabismus can be determined. These methods are easier to interpret and require less computational power, allowing for fine-tuning at each stage. However, their performance heavily depends on the quality and relevance of manually extracted features and may struggle with high-dimensional data and complex patterns inherent in ocular position photos. Additionally, the selection of thresholds based on limited statistical data within smaller datasets may introduce bias, reducing the model’s generalizability.

In contrast, end-to-end deep learning algorithms, such as convolutional neural networks (CNNs) and recurrent neural networks (RNNs), learn features and patterns directly from raw data, automating the feature extraction process. These models use extensive image datasets to enhance the model’s generalization capability, handling large, high-dimensional datasets and capturing complex, non-linear relationships within the data. This leads to higher accuracy and robustness in predictions. However, they require significant computational resources and large amounts of labeled data for training, and their “black-box” nature can make interpretation and troubleshooting more challenging.

In conclusion, end-to-end deep learning models generally outperform traditional machine learning algorithms in accuracy and generalizability due to their ability to learn directly from data without manual feature extraction. Deep learning models scale better with increased data, improving performance as more data becomes available, whereas traditional methods may not show the same level of improvement due to the limitations of manual feature extraction. While traditional machine learning models are more interpretable, providing clearer insights into the decision-making process, deep learning models offer superior performance and scalability for strabismus diagnosis using ocular position photos. The choice between the two depends on the specific requirements and constraints of the clinical application, including the availability of computational resources and the need for model interpretability. This comparative understanding highlights the potential of integrating both approaches to optimize diagnostic accuracy and clinical applicability.

### AI in strabismus surgical planning and prognostication

The application of AI in strabismus surgical planning and prognostication represents a transformative shift towards precision medicine in pediatric ophthalmology. Recent studies exemplify the integration of advanced machine learning models, such as Support Vector Regression (SVR) [37], multi-output regression trees [38], Light Gradient Boosting Machines (LightGBM) [34], convolutional neural networks (CNN) [39], and recurrent residual CNNs [40] with global attention gates, to refine surgical strategies for complex ocular deviations. These AI-driven tools process extensive clinical data, including visual acuity, type of deviation, binocular fixation, and ocular position, to predict the precise amounts of muscle resection and recoil needed in surgeries.

De Almeida et al. [32] conducted a regression study on 88 patients with horizontal strabismus using Support Vector Regression (SVR). The model aimed to accurately estimate the adjustments needed for medial and lateral rectus muscles. The SVR model achieved mean absolute errors of 0.5 mm for medial rectus recoil and 0.7 mm for resection, with 0.6 mm for lateral rectus recoil and 0.8 mm for resection [32]. Leite et al. [33] analyzed surgical planning for strabismus in 153 patients with horizontal deviations, primarily esotropia and exotropia. Utilizing comprehensive patient data including age, binocular fixation, and deviation from five ductions positions, the study applied a multi-output regression tree approach to predict necessary surgical adjustments for various muscles. The model accurately forecasted recoil and resection requirements for both medial and lateral rectus muscles, achieving a Mean Absolute Error (MAE) range of 0.448 mm–1.038 mm and a Root Mean Square Error (RMSE) of up to 1.496 mm [33]. Tang et al. [34] conducted a study involving 1,076 surgical cases of patients with various types of strabismus, including esotropia and exotropia, using patient data like age, dominant eye, and exodeviation angles. They developed a model using the Light Gradient Boosting Machine (LightGBM) architecture, which initially achieved a 69.32% accuracy in predicting surgical adjustments such as muscle movement amount and direction. To address data scarcity and improve model performance, the Wasserstein Generative Adversarial Network with Gradient Penalty (WGAN-GP) was applied, enhancing accuracy to 84.52% [34]. Mao et al. [29] developed an Operation Advice System using corneal light-reflection photos from 56 exotropia patients who had successfully undergone initial surgeries. The retrospective test set included 160 subjects, predominantly with intermittent exotropia, and demonstrated a high positive correlation (r = 0.86, *P* < 0.001) between the predicted and actual surgical angles, with an accuracy of ±5.5° (11.5 PD) and a slight bias of −0.6° [29]. Lou et al. [35] developed a recurrent residual CNN based on GAR2U-Net to evaluate Inferior Oblique Overaction (IOOA) in a study involving 106 eyes of 72 patients. The technique focused on measuring the height difference between the inferior corneal limbus of both eyes, a key indicator of IOOA, which was clinically graded from +1 to +4. The study found significant correlations between automated photographic measurements and clinical gradings, and excellent agreement with manual measurements, indicated by intraclass correlation coefficients (ICCs) of 0.975. This study demonstrates advanced neural networks’ ability to offer cost-effective, accurate, and scalable IOOA assessments using simple photographs [35]. Liu et al. [36] conducted a prospective cross-sectional study on 132 patients with intermittent exotropia, using a support vector machine (SVM) to determine the optimal timing for surgical interventions. The study utilized preoperative factors like deviation angle, binocular vision, and stereoacuity to predict the best moment for surgery. Post a 6-month follow-up, the SVM achieved an 82.1% accuracy rate, with an overall success rate of 63.6%, identifying the initial postoperative deviation angle as a crucial predictor of successful alignment. This research highlights the effectiveness of SVM in enhancing surgical outcomes for exotropia through precise timing based on detailed clinical data [36].

The aforementioned study illustrate that AI integration refines surgical planning and prognostication. By leveraging these technologies, ophthalmologists can achieve significantly improved accuracy in surgical outcomes, enhancing patient-specific treatment plans. Notably, these models facilitate the analysis of deviations, muscle actions, and postoperative alignments, allowing for highly individualized surgical interventions. This marks a significant shift in enhancing surgical precision and redefining strabismus management standards.

## Discussion

The integration of artificial intelligence (AI) in strabismus management represents a significant advancement in ophthalmology, enhancing diagnostic precision and therapeutic efficacy. As AI technologies have begun to permeate every aspect of healthcare, from data collection and integration [41–45] to analysis and treatment [46], they demonstrate a potent synergy with medical professionals, showcasing their immense potential to enhance human health [47]. The advent of large models like ChatGPT has marked a pivotal shift, increasingly evident in the fourth industrial revolution, driving transformative changes in how healthcare is delivered.

While AI’s application in healthcare, particularly in ophthalmology, is promising, it faces challenges such as the need for improved data-sharing mechanisms, validation, evaluation, and regulatory frameworks. Despite the impressive achievements of AI in enhancing strabismus screening, diagnosis, and treatment, there are significant limitations regarding the representativeness and generalizability of these advancements in real-world clinical settings. Current research often excludes numerous strabismus types, such as vertical, restrictive, sensory, paralytic strabismus, myasthenia gravis, nystagmus, and Duane syndrome, focusing primarily on pediatric populations and concomitant strabismus patients. This oversight highlights a critical gap, as there is a pressing need for comprehensive screening and precise diagnostic processes that encompass all age groups and subtypes of strabismus. Thus, the utility of these AI models in diverse clinical environments remains questionable. Meanwhile, despite early advantages in AI research facilitated by the digitization in ophthalmology, which amassed extensive imaging and pathological data, the comprehensive integration of multimodal patient data remains scarce, and the reliance on single data sources for AI diagnostics and prognostic assessments presents substantial challenges. Typical methodologies primarily use ocular position photos or eye movement videos for diagnosing strabismus, or electronic health records for predicting surgical outcomes, without considering additional data inputs, such as the sensory exam results, the refractive status, the CT or MRI images, forced duction test, etc. Conversely, clinicians often employ a multimodal data approach, integrating those concurrent data streams to enhance the accuracy of disease diagnosis, prognostic evaluations, and therapeutic strategies. Current AI systems process static, singular time-point data, which fails to capture the dynamic, evolving nature of diseases and overlooks the rich historical context of patient data that could significantly refine predictive accuracies. Theoretically, as AI technologies advance, models should be capable of harnessing all available data sources, including those traditionally inaccessible to clinicians, such as genomics [48]. Moreover, in regions such as China, many hospitals still rely on non-digital, unstructured data collection methods due to a lack of digital resources and personnel, leading to incomplete or inaccurate medical data and hindering widespread AI research. Additionally, the field suffers from a scarcity of AI-driven strabismus management products that extend beyond diagnosis to include essential services such as patient referral and long-term follow-up, which are vital for ensuring sustained care and systematic data collection across various modalities and time points.

Moving forward, research utilizing deep learning in strabismus should embrace a more inclusive approach, incorporating patients of all ages, ethnicities, and all types of the condition. However, the diversity in irrelevant features such as eyelid size, eye color, and skin tone across different age groups and ethnic backgrounds poses additional challenges for model training. Complications such as eyelid ptosis, which require physical assistance during photo acquisition, can disrupt the accurate extraction of features by AI models. Additionally, ensuring privacy protection is critical as AI technologies handle sensitive medical data. Addressing these challenges is essential for maximizing the clinical utility of AI technologies. Future initiatives should focus on easily collectible multimodal data for strabismus research, integrating biosensors, electronic medical records, eye movement videos, ocular photos, and socio-environmental factors throughout the patient care timeline. Large AI models, such as Transformer-based architectures, have the potential to handle the complexity and scale of multimodal data, offering enhanced generalization and predictive power. This integration not only facilitates extensive research but also enables the construction of ophthalmological knowledge graphs and the provision of personalized treatment recommendations, which are becoming increasingly feasible. Meanwhile, the development of an integrated AI-based strabismus management platform that facilitates paperless, comprehensive management of strabismus patients from screening through treatment, and enables the integration and collection of cross-modal patient information, will lay a solid foundation for a specialized, large-scale AI model in strabismus care. The platform should support AI applications in the screening, diagnosis, surgical parameter estimation, and prognosis prediction of strabismus, as well as intelligent follow-up. By leveraging a full-process, multimodal, and multi-timepoint intelligent platform, ophthalmologists can achieve more accurate, personalized, and effective strabismus management, ultimately improving patient outcomes and advancing the field of ophthalmology.

## Conclusion

In conclusion, this review has highlighted significant advancements and emerging challenges in integrating AI into strabismus management. AI is revolutionizing screening, diagnosis, and surgical planning through cutting-edge technologies such as deep learning, enhancing accuracy and optimizing outcomes. However, critical gaps are evident, particularly the underrepresentation of diverse strabismus types and age groups in AI studies. These limitations underscore the necessity for AI systems that accommodate broader demographics and clinical scenarios. Additionally, the reliance on single-source data in AI models, compared to the multimodal approach used in clinical settings, reveals an urgent need for integrating more comprehensive data sources, including longitudinal patient data. Addressing these challenges will be pivotal in advancing AI technology in ophthalmology, enhancing patient outcomes, and setting new standards in healthcare delivery.
